# Databases of ligand-binding pockets and protein-ligand interactions

**DOI:** 10.1016/j.csbj.2024.03.015

**Published:** 2024-03-24

**Authors:** Kristy A. Carpenter, Russ B. Altman

**Affiliations:** a Department of Biomedical Data Science, Stanford University, Stanford, CA 94305, USA; b Department of Bioengineering, Stanford University, Stanford, CA 94305, USA; c Department of Genetics, Stanford University, Stanford, CA 94305, USA; d Department of Medicine, Stanford University, Stanford, CA 94305, USA

**Keywords:** Binding sites, Protein-ligand interaction, Pocket detection, Databases, Protein structure, Structural bioinformatics

## Abstract

Many research groups and institutions have created a variety of databases curating experimental and predicted data related to protein-ligand binding. The landscape of available databases is dynamic, with new databases emerging and established databases becoming defunct. Here, we review the current state of databases that contain binding pockets and protein-ligand binding interactions. We have compiled a list of such databases, fifty-three of which are currently available for use. We discuss variation in how binding pockets are defined and summarize pocket-finding methods. We organize the fifty-three databases into subgroups based on goals and contents, and describe standard use cases. We also illustrate that pockets within the same protein are characterized differently across different databases. Finally, we assess critical issues of sustainability, accessibility and redundancy.

## Graphical abstract

**Figure:**
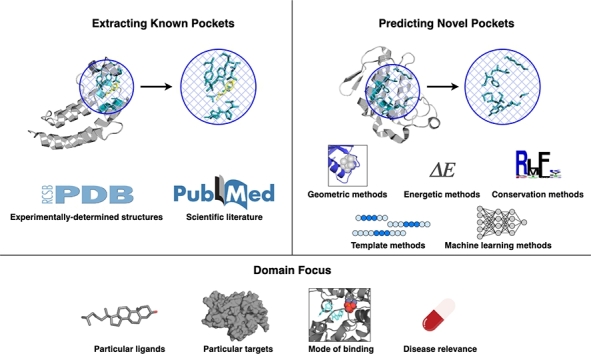

## 1 Introduction

In an age of remarkable advances in artificial intelligence (AI) and subsequent AI-powered advances in biomedical research [1], [2], [3], it is abundantly clear that computational methods, particularly machine learning (ML) models, can make the notoriously slow and expensive [4], [5], [6], [7], [8] process of drug discovery and development more efficient. Two oft-given examples of how computation can aid drug discovery are those of virtual screening and drug repurposing. Virtual screening [9], [10], [11], [12] is an *in silico* alternative to traditional high-throughput screening, in which a large quantity of compounds are assayed for desired activity. While an *in vitro* high-throughput screen requires obtaining all tested compounds and physically running assays, a virtual screen can be conducted faster and more cheaply to identify compounds with higher likelihood of *in vitro* and *in vivo* success. Drug repurposing, also called drug repositioning, is the concept of taking a compound developed for one indication and applying it to another [13], [14]. Repurposing a drug or drug lead that passed initial safety screenings allows for quicker development than designing a novel compound; this is desirable in scenarios where a new therapeutic is needed imminently, as in the COVID-19 pandemic [15], [16]. Many virtual screening and drug repurposing methods require knowledge of the locations at which a drug may bind to a protein in order to compute the likelihood of the drug having an effect. These locations, called binding sites or binding pockets, can be determined experimentally or computationally. There are many machine learning methods for virtual screening [17], [18], [19] and drug repurposing [20], [21]. For such data-hungry methods, and large-scale discovery generally, it is useful to have a precomputed database of binding pockets as opposed to identifying pockets on-the-fly. Such databases make a wealth of pocket information quickly and easily accessible to all researchers, including those without computational expertise.

The need for efficient manners of identifying pockets is made more pressing due to the development of protein structure prediction methods that have achieved accuracy on par with experiment [1], [2], [22] and subsequent proteome-scale databases of predicted structure. While the Protein Data Bank (PDB) [23], the central source of experimentally-determined protein structures, contains approximately 215,000 structures as of early 2024, the AlphaFold Protein Structure Database [24] and ESM Metagenomic Atlas [2] each contain hundreds of millions of structures. This vast increase in protein structure data is driven by the ability of methods like AlphaFold2 [1] and ESMFold [2] to close the gap between the amount of sequence and structure data available across many proteomes. More data enables larger-scale and more powerful analyses, but only if there are methods and resources to efficiently extract biologically-pertinent information.

There are many existing pocket databases. It is important to have an up-to-date overview of these different databases, their capabilities, their contents, and their accessibility to facilitate work leveraging them and to protect against redundant efforts. Such reviews have been written previously [25], [26], [27], but as new databases emerge and old databases cease to be maintained, a new survey is required. Here, we present lists of currently available databases (as of early 2024) with binding pocket information and protein-ligand interaction information, organized into subgroups and with brief descriptions of each. With 53 databases described (37 pocket databases and 42 interaction databases), we present a broader set of such databases than has been previously described.

This review is structured as follows: in Section 2 (“What Is A Pocket?”), we discuss the problem of defining a ligand-binding pocket and the common approaches to doing so; in Section 3 (“Pocket-Finding Methods”), we provide an overview of algorithms for identifying pockets; in Section 4 (“Pocket Databases”), we describe 37 pocket databases and organize them into subgroups; in Section 5 (“Interaction Databases”), we describe 16 interaction databases and organize them into subgroups; in Section 6 (“How Are These Databases Used?”), we explain different uses for pocket and interaction databases and provide real-world examples of previous usage from the literature; in Section 7 (“Comparison of Database Contents”), we provide four proteins as case studies to illustrate how different databases represent pockets and give a further example of how to apply these databases for early-stage drug development; and we conclude in Section 8 (“Discussion”) with discussion of emergent themes and recommendations for future use and creation of pocket and interaction databases.

## 2 What is a pocket?

There is not one standard definition of a “pocket” used uniformly across pocket-finding methods or pocket databases. It is important to understand the distinctions between these definitions for effective usage of these resources. We first establish that the domain of this review is ligand-binding pockets – that is, areas on the surface of a protein which bind small molecules. Most small molecule binding sites are concave and hydrophobic. Protein surfaces that bind other proteins are inherently different due to their larger, flatter, and less hydrophobic surfaces [28], [29], [30]; this is outside the scope of our review, and we direct the interested reader to recent reviews on protein-protein interaction (PPI) interface prediction [31], [32], [33].

A common definition of a binding pocket seen when working with structures of experimentally derived protein-ligand complexes is to select a cutoff distance and designate all residues within that cutoff distance from any ligand atom to be part of the binding pocket. This cutoff distance is typically around 5Å. A modified version of this criteria comes from the BioLiP database [34]: the cutoff for inclusion in the binding pocket depends upon the sum of the radii of the closest pair of residue and ligand atoms. These distance-based definitions make the pocket easy to compute given a structure coordinate file. However, defining the pocket based on distance to the ligand is not fully accurate to the mechanism of ligand binding. The thermodynamics of protein-ligand binding is complex (no pun intended), and it is not obvious which residues are involved in ligand binding, or in which conformation they bind [35], [36]. The set of residues whose interactions with the ligand are required for binding can be investigated experimentally by functionally characterizing catalytic residues, alanine scanning, or heteronuclear-NMR-based screening [37], [38], [39]. As it is infeasible to conduct such experiments for as many proteins as we have an experimentally-determined structure for, approximating the binding pocket with the distance-based criteria yields the most data with minor losses in accuracy.

In the prediction setting, there is not a ligand around which to define the pocket, so the criteria for determining which residues constitute the pocket changes. There are different types of pocket prediction methods (described in Section 3, below) and the nature of the predicted pocket is related to the pocket prediction strategy. For example, methods based on 3D protein structure that have a geometric component (geometry-based, energy-based, some template-based methods, and structure-based machine learning methods, described further below) will typically predict pockets that are sets of residues clustered in 3D structure space. Methods based on sequence (conservation-based, some template-based methods, and sequence-based machine learning methods, described further below) will typically predict pockets that are sets of residues proximal to each other in the primary sequence. These two different types of pockets will visually appear different and it is difficult to compare performance of methods that do not produce the same type of pocket.

Besides the amino acids of the protein that constitute the binding pocket, water molecules also play a key role in binding pocket dynamics. Case studies on different proteins have shown that the way in which water is accounted for results in changes in computational predictions of ligand binding [40], [41] and experimental structural characterizations of binding pockets have revealed the mechanistic details of the contributions of water in different proteins [42], [43]. Therefore, when considering a binding pocket, it is important to include the solvent in addition to the protein surface. While further discussion of the role of water in ligand binding is out of scope of this review, we direct the interested reader to existing work that does address this [44], [45], [46], [47], [48].

We emphasize that all the different pocket definitions mentioned here are relevant to different contexts. It is important to consider which definition is appropriate for one's use case when selecting a pocket-finding method or database, as well as to ensure that any comparisons between different pocket-finding methods have consistent pocket definitions to avoid inadvertently biasing the evaluation toward a particular method [49].

A related term that is important to define is “druggability,” as the stated motivation for the majority of pocket databases or pocket-finding methods is to provide “druggable” pockets for use in therapeutic development. Druggability is not always consistently defined. In some contexts, it is meant to denote the ability to bind a (often drug-like) ligand, whereas in others it means the ability for its binding to affect downstream disease-related function. To distinguish between these two notions, we will here only use the term “ligandability” to denote the former [50], and only use the term “druggability” to denote the latter.

## 3 Pocket-finding methods

Besides databases, there exists a glut of algorithms that identify potential pockets on the surface of a protein (Table S1). While not the focus of this review, we will briefly provide an overview of pocket-finding algorithms to give background necessary to appreciate the databases described below.

The majority of pocket-finding methods can be categorized into one of five different approaches: geometry-based, energy-based, conservation-based, template-based, or machine learning-based. There are also consensus methods that use a combination of these approaches. Some of the listed methods are blind docking programs, which both identify binding pockets on the surface of a query protein and predict the pose of a query ligand in the binding pocket. While blind docking methods may at times not be included in lists of pocket-finding methods as they do not only serve to identify pockets, we do so here to highlight the methods underlying their initial pocket-finding components.

This overview of methods is not exhaustive. We refer the interested reader to recent reviews on pocket-finding methods for a more in-depth discussion [25], [51], [52], [53], [54], [55], [56], [57].

### 3.1 Geometry-based

The driving idea behind geometry-based pocket-finding methods is that most binding pockets are concave regions on the protein surface into which a ligand can fit. Broadly, these methods all use geometric criteria to detect cavities on the protein. They typically accomplish this by identifying small vacant spaces on the surface of the protein and clustering these small cavities together into pockets. There are three predominant strategies for the first stage of identifying small vacant cavities: constructing a Voronoi tessellation over the protein surface space and taking its vertices as cavities; constructing a discrete grid and taking grid cells that do not clash with the protein as cavities; and constructing probe spheres to fill the surface of the protein and taking each of those spheres as cavities [55]. Some examples of geometry-based pocket-finding methods are CB-Dock [58] (spheres), CAVITY [59] (spheres), CASTp [60] (Voronoi), DoGSiteScorer [61] (grid), fpocket [62] (Voronoi), LigandFit [63] (grid), LIGSITE [64] (grid), MolDock [65] (grid), SiteFerret [66] (spheres), and SURFNET [67] (spheres).

### 3.2 Energy-based

Energy-based pocket-finding methods are characterized by their use of energy functions to determine the most favorable sites for binding. The energy functions used often originate from molecular dynamics (*e.g.* CHARMM [68], OPLS-AA [69], AMBER [70]). Many energy-based methods also take geometry of the protein surface into account. Another similarity with geometry-based methods is that energy-based methods frequently use a grid or probe sphere approach to determine points at which to evaluate the energy function. Some examples of energy-based pocket-finding methods are FTMap [71], GRID [72], Q-SiteFinder [73], and SiteMap [74].

### 3.3 Conservation-based

Binding pockets play a critical role in protein function, making them likely to be evolutionarily conserved. Conservation-based pocket-finding methods leverage this reasoning to predict pockets. Predicting pockets based on their degree of evolutionary conservation requires evolutionary information, often in the form of a multiple sequence alignment (MSA). Unlike geometry- and energy-based methods, they do not require a 3D structure of the query protein. Some examples of conservation-based pocket-finding methods are Capra and Singh's information theoretic approach [75] and Conseq [76].

### 3.4 Template-based

Template-based pocket-finding methods are similar to conservation-based methods in that they make use of the context of evolution to predict pockets. The line of reasoning underlying template-based pocket-finding is that proteins have common origins, so different proteins may share common motifs. The sequence or structure of the query protein is therefore checked against a library of known binding site templates; if a segment of sequence or structure has high similarity to a template, then it is predicted as a binding site. Some examples of template-based pocket-finding methods are 3DLigandSite [77] (structure templates), COACH [78] (sequence and structure templates), COFACTOR [79] (structure templates), and eFindSite [80] (structure templates).

### 3.5 Machine learning-based

Machine learning has existed since the 1950s [81], but recent breakthroughs in deep learning methods, improvements in available hardware, and increases in protein-related data have led to the creation of many ML-based pocket-finding methods in the past several years. Similar to template-based methods, the inputs to ML-based methods can be protein sequences or structures. These different inputs benefit from different inductive biases; sequence-based ML methods tend to use architectures commonly used for 1D and 2D data such as convolutional neural networks and transformers, whereas structure-based ML methods tend to use architectures commonly used for 3D data such as graph neural networks and equivariant neural networks. Some examples of ML-based pocket-finding methods are DeepSite [82] (structure), DeepSurf [83] (structure), EQUIBIND [84] (structure), HoTS [85] (sequence), MaSIF [86] (structure), MFR-DTA [87] (sequence), PocketMiner [88] (structure), and PRANK [89] (structure).

### 3.6 Consensus

Consensus methods take an ensemble approach, with the motivation that there are advantages and disadvantages to each of the different types of pocket-finding methods, and that using a combination of methods will improve performance. CB-Dock2 [90] combines the geometry-based CB-Dock with a template-based pipeline and outputs the top scoring sites. Three different methods combine a geometry-based method with sequence conservation criteria: SURFNET-Consurf [91], LIGSITEcsc [92], and ConCavity [93]. MetaPocket [94] combines eight different methods – six geometry-based (fpocket, LIGSITE, SURFNET, GHECOM [95], PASS [96], POCASA [97]), one energy-based (Q-SiteFinder), and one consensus (ConCavity).

## 4 Pocket databases

We assembled a list of relevant databases from previous review papers [25], [26], [27], [98], [99], [100], [101] and prior domain knowledge. To identify newer databases and to minimize the number of relevant databases neglected, we also searched PubMed with the following query:

(“binding site”[Title/Abstract] OR “protein-ligand interaction”[Title/Abstract] OR “drug-target interaction”[Title/Abstract] OR “binding pocket”[Title/Abstract] OR “ligand binding”[Title/Abstract] OR “small molecule binding”[Title/Abstract]) AND (“database”[Title/Abstract] OR “atlas”[Title/Abstract] OR “db”[Title/Abstract] OR “resource”[Title/Abstract])

We manually checked titles and abstracts of all returned results for relevance to this review.

**Fig. 1:**
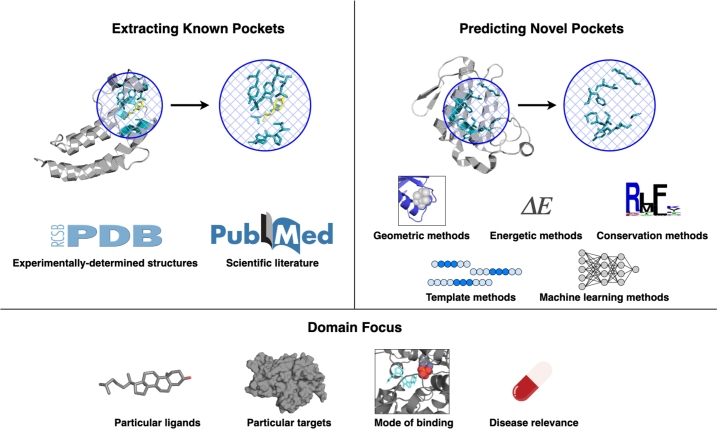
Schematic depicting the different kinds of pocket databases covered by this review. Each database can have a different mode of extracting known pockets, predicting novel pockets, and/or a specific domain focus.

We define a “pocket database” as a database that designates which residues of a protein are part of a ligand binding pocket or provide a coordinate for the center of such a pocket with the corresponding structure file as context. From our assembled database list, we identified a total of 58 pocket databases adhering to this definition (Table S2), 37 of which have a website or download available at the time of writing (Table 1). These databases have different focuses and are useful for different applications; we group them into subcategories (Fig. 1).

**Figure:**
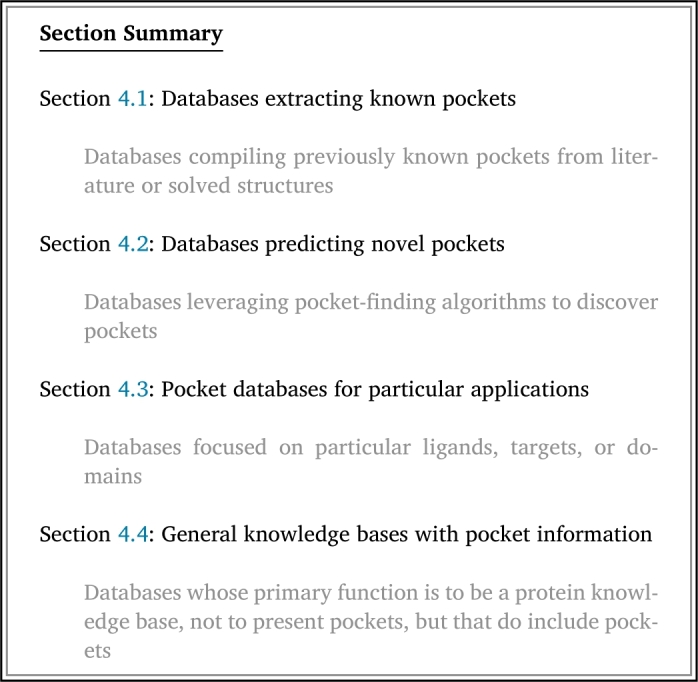

**Table 1:** Details for all pocket databases described in this review, along with most recent reference and active URL as of March 2024. Labels correspond with subsection in which databases are described in the body of the review. A “browsable” website refers to a website on which the user can explore the contents of the database without downloading files or using an Application Programming Interface (API). A database is denoted with “download available” if the entirety of the database is available to download at once. See Table S2 for more information.

Name	Year Created	Last Updated	Labels	Browsable Website Available	Download Available	API Available	URL
AroCageDB [110]	2021	2021	For particular applications;	yes	yes	no	https://drug-discovery.vm.uni-freiburg.de/arocagedb/
			Particular mode of binding				
ASD [111]	2011	2023	For particular applications;	yes	yes	no	https://mdl.shsmu.edu.cn/ASD/
			Particular mode of binding				
Binding MOAD [112]	2005	2023	Extracting known pockets;	yes	yes	no	http://www.bindingmoad.org/
			Biologically-relevant only				
BioLiP [34]	2013	2024	Extracting known pockets;	yes	yes	yes	https://seq2fun.dcmb.med.umich.edu//BioLiP/index.cgi
			Biologically-relevant only				
CASF [113]	2007	2016	Extracting known pockets;	no	yes	no	http://www.pdbbind.org.cn/casf.php
			Docking benchmark				
CASTp [60]	2003	2018	Predicting novel pockets;	yes	no	no	https://sts.bioe.uic.edu/castp/
			Whole PDB				
CaviDB [114]	2022	2022	Predicting novel pockets;	yes	no	no	https://www.cavidb.org/
			Whole human proteome				
CavitySpace [115]	2022	2022	Predicting novel pockets;	yes	yes	no	http://www.pkumdl.cn:8000/cavityspace/
			Whole human proteome				
DeepCholesterol [116]	2018	2018	For particular applications;	yes	no	no	https://deepcholesterol.soton.ac.uk/
			Particular ligands				
eF-site [104]	2001	2024	Extracting known pockets;	yes	no	no	https://pdbj.org/eF-site/
			Whole PDB				
eModel-BDB [117]	2018	2018	Predicting novel pockets;	yes	yes	no	https://brylinski.org/emodel-bdb-0
			Known binding affinity				
FireDB [118]	2007	2020	Extracting known pockets;	yes	yes	yes	https://firedb.bioinfo.cnio.es/Php/FireDB.php
			Biologically-relevant only				
G2P Portal [119]	2023	2023	Protein knowledge base	yes	no	yes	https://g2p.broadinstitute.org/
Het-PDB Navi [107]	2004	2024	Extracting known pockets;	yes	no	no	https://hetpdbnavi.nagahama-i-bio.ac.jp/
			Whole PDB				
HProteome-BSite [120]	2023	2023	Predicting novel pockets;	yes	yes	no	https://galaxy.seoklab.org/hproteome-bsite/database/
			Whole human proteome				
InterPro [121]	1999	2024	Protein knowledge base	yes	yes	yes	https://www.ebi.ac.uk/interpro/
KLIFS [122]	2014	2024	For particular applications;	yes	yes	yes	https://klifs.net/
			Particular targets				
LigBase [108]	2002	2003	Extracting known pockets;	yes	no	no	https://modbase.compbio.ucsf.edu/ligbase/
			Whole PDB				
MbPA [123]	2023	2023	For particular applications;	yes	yes	yes	http://bioinfor.imu.edu.cn/mbpa/
			Particular ligands				
MetalPDB [124]	2013	2023	For particular applications;	yes	yes	yes	https://metalpdb.cerm.unifi.it/
			Particular ligands				
PDBSite [103]	2000	2014	Extracting known pockets;	yes	no	no	http://wwwmgs.bionet.nsc.ru/mgs/gnw/pdbsite/
			Whole PDB				
PDBSpheres [109]	2022	2022	Extracting known pockets;	no	yes	no	https://github.com/LLNL/PDBspheres
			Whole PDB				
PDBsum [106]	1997	2023	Extracting known pockets;	yes	yes	no	https://www.ebi.ac.uk/thornton-srv/databases/pdbsum/
			Whole PDB				
PDID [125]	2014	2015	For particular applications;	yes	yes	no	http://biomine.cs.vcu.edu/servers/PDID/index.php
			Drug- and disease-relevance				
Pharos [126]	2016	2023	For particular applications;	yes	yes	yes	https://pharos.nih.gov/
			Drug- and disease-relevance				
PocketQuery [127]	2011	2017	Predicting novel pockets;	yes	no	no	http://pocketquery.csb.pitt.edu/
			Whole PDB				
PrankWeb3 [128]	2019	2022	Predicting novel pockets;	yes	yes	yes	https://prankweb.cz/
			Whole human proteome				
PrePCI [129]	2023	2023	Predicting novel pockets;	yes	no	no	https://honiglab.c2b2.columbia.edu/prepci.html
			Whole human proteome				
ProBiS-Dock [130]	2021	2021	Extracting known pockets;	yes	yes	no	http://probis-dock-database.insilab.org/
			Docking benchmark				
ProBiS-Fold [131]	2022	2022	Predicting novel pockets;	yes	yes	no	http://probis-fold.insilab.org/
			Whole human proteome				
PROCARB [132]	2008	2016	For particular applications;	no	yes	no	https://www.procarb.org/procarbdb/
			Particular ligands				
Q-BioLiP [133]	2023	2024	Extracting known pockets;	yes	yes	no	https://yanglab.qd.sdu.edu.cn/Q-BioLiP/
			Biologically-relevant only				
sc-PDB [102]	2004	2017	Extracting known pockets;	yes	yes	no	http://bioinfo-pharma.u-strasbg.fr/scPDB/
			Whole pdb				
SIFTS [134]	2002	2024	Protein knowledge base	no	yes	yes	https://www.ebi.ac.uk/pdbe/docs/sifts/
TTD [135]	2002	2024	For particular applications;	yes	yes	no	https://db.idrblab.net/ttd/
			Drug- and disease- relevance				
UniProt [136]	2003	2024	Protein knowledge base	yes	yes	yes	https://www.uniprot.org/
ZincBind [137]	2019	2022	For particular applications;	yes	no	yes	https://zincbind.net/
			Particular ligands

### 4.1 Databases extracting known pockets

#### 4.1.1 Databases extracting known pockets: whole PDB

**sc-PDB**[102] is a database of small molecule pockets in the PDB, created for the purpose of screening potentially ligandable pockets in structure-based drug discovery. sc-PDB was built by extracting all PDB entries with small molecule ligands with molecular weight between 140Da and 800Da that are not cofactors or solvent. On the sc-PDB website, users can search the database by protein, ligand, binding mode (*e.g.* specifying the number of hydrophobic contacts or hydrogen bond donors), or binding site (*e.g.* specifying cofactors, number of water molecules, or site size). Each sc-PDB entry contains information about the binding pocket and similarity calculations based on binding site, cavity, and binding mode to enable discovery of similar pockets.

**PDBSite**[103] is a database of protein functional sites, including but not limited to binding pockets, extracted from the PDB. Besides small molecule ligand binding sites, PDBSite also includes DNA binding sites, RNA binding sites, PPI sites, and catalytic sites. Sites included in PDBSite are obtained by processing the metadata text included in each PDB entry in addition to identifying residues proximal to listed heteroatoms.

**eF-site (electrostatic-surface of Functional site)**[104] is also a database of protein functional sites and is affiliated with the Protein Data Bank Japan (PDBj). eF-site augments the structural information of these sites already present in the PDB with calculations of their electrostatic surfaces. Electrostatics of a protein site can explain its function and determine which types of ligands it can bind. eF-site organizes functional sites into categories including binding sites, catalytic sites, motifs identified by PROSITE pattern, modified residues, transmembrane domains. On the eF-site website, users can visualize the electrostatic surface using the Molmil viewer [105].

**PDBsum**[106] was developed to summarize and enhance interpretation of all PDB entries, including but not limited to those with protein-ligand interactions. For a given PDB entry with small molecule ligands, PDBsum provides ligand validation and per-residue interactions. PDBsum entries also have a “Clefts” section, which highlights binding sites on the protein surface and provides basic properties such as site volume and average depth.

**Het-PDB Navi**[107] is a database of small molecule interactions present in the PDB. In addition to providing information on ligands and interacting residues for each included PDB structure, Het-PDB Navi also allows the user to browse for PDB structures containing a given ligand of interest.

**LigBase**[108] is another database containing interactions between proteins and small molecules in the PDB; the primary purpose of LigBase is to provide structural alignments between binding sites and structural templates from the PDB. The suggested use of LigBase is to hypothesize that sites with high structural similarity to a known binding site may bind the same ligand, which can be applied for drug repurposing.

**PDBSpheres**[109] is a method for identifying novel binding sites by using structural similarity to known binding sites; its library of known binding sites is publicly available and is included in this review as another pocket data repository. The library was created by obtaining every structure with a ligand (including peptide, metal, and ion ligands) from the PDB and creating a sphere microenvironment of every residue with at least one atom within 12Åof the ligand.

#### 4.1.2 Databases extracting known pockets: biologically-relevant binding

**BioLiP**[34] contains all PDB entries with biologically-relevant ligand binding interactions. While the PDB specifies the biological assembly, which is the functional structure of the molecule as opposed to the full repetitive crystal structure or the smallest asymmetric unit, this biological assembly still includes all heteroatoms present in the crystal structure. This often includes purification and crystallization additives, which are artifacts of the structure determination process and not biologically-relevant. BioLiP aims to include only biologically-relevant ligands, including metals and nucleic acids in addition to small molecules. The curation process for biologically-relevant binding is semi-manual and its code base is open-source.

Each entry in BioLiP contains information about the protein and ligand, binding residues, and any catalytic site residues. Both binding residues and catalytic site residues are provided with both the original PDB numbering scheme and a reindexed scheme that corrects for insertions or gaps in the PDB file. On the BioLiP website, users can search for entries by name of protein or ligand, sequence, or structure.

**Q-BioLiP**[133] is an extension of BioLiP that purports to address some of the problems with BioLiP's data and curation process. BioLiP represents protein structures as single-chain tertiary structures; this neglects quaternary structure. Q-BioLiP's primary contribution is representing protein quaternary structure in its entries, capturing ligand interactions that involve multiple chains simultaneously. Q-BioLiP makes small modifications to the curation criteria used by BioLiP, and in addition contains both biologically-relevant and biologically-irrelevant interactions (with both respectively labeled), with the justification that falsely labeling an interaction as biologically-irrelevant leads to data loss and that users may differ in their assessment of biological relevance from the curators. Q-BioLiP also distinguishes itself from BioLiP with respect to binding affinity. BioLiP only provides experimentally determined binding affinities obtained from Binding MOAD, PDBbind, BindingDB, or direct literature search; Q-BioLiP provides these as well as computationally predicted binding affinities for any entry without experimentally determined binding affinity.

**Binding MOAD**[112] contains all interactions between proteins and biologically relevant ligands with resolution of at least 2.5Åpresent in the PDB. Like BioLiP, Binding MOAD contains binding affinity values for entries if present in the literature. Binding MOAD additionally contains homology families, 2D and 3D similarity calculations between ligands, and similar binding sites. While Binding MOAD has seen frequent use throughout its lifetime, its website is scheduled to cease being available starting July 2024.

**FireDB**[118] is another database of biologically-relevant ligand interactions in the PDB. It is focused on providing annotations of residues with functional importance. FireDB classifies each instance of protein-ligand binding in the PDB as either cognate (naturally-occurring, endogenous ligands), non-cognate (ligands determined to be non-biologically-relevant / naturally-occurring), or ambiguous (ligands that can be cognate or non-cognate depending on context). FireDB also contains firestar, a sequence- and structure-alignment-based method for predicting functional residues, including binding sites.

#### 4.1.3 Databases extracting known pockets: docking benchmarks

The **CASF (Comparative Assessment of Scoring Functions)** dataset [113] is a subset of PDBbind (described in Section 5) intended to be a benchmark for scoring functions used in docking. CASF was created by selecting high-quality PDBbind structures, clustering them by sequence identity, and including representative protein-ligand complexes from each cluster with a wide range of binding affinity values. The dataset contains the selected protein structures without ligand, the ligand in its native conformation with coordinates that can be used to identify the pocket on the protein structure, decoy conformations, and experimentally-determined binding affinities.

The **ProBiS-Dock Database** [130] was also primarily intended for use with docking, and contains all small molecule binding sites present in the PDB. The ProBiS-Dock Database is distinct from other similar databases in that it retains cofactors and other accessory ligands at the binding site, as they influence substrate binding. ProBiS-Dock also allows binding sites to include multiple chains. Each binding site entry includes a list of binding residues, accessory ligands, and a ligandability score. The ligandability score is an estimate of how likely the binding site is to bind a drug-like compound and does not capture druggability in the sense of functional modulation. Besides known binding sites and ligands from the PDB, the ProBiS-Dock database also provides binding sites predicted by the ProBiS-Dock algorithm [138] (a structural template method) and potential ligands predicted by binding site similarity.

### 4.2 Databases predicting novel pockets

#### 4.2.1 Databases predicting novel pockets: whole PDB

**CASTp (Computed Atlas of Surface Topography of proteins)**[60] is a web server that contains pocket predictions for all PDB structures that are designated as biologically significant. Pocket predictions are made using a geometric algorithm that leverages Voronoi tessellation. Besides listing residues in each predicted pocket, CASTp also provides per-residue functional annotations from UniProt and SIFTS.

**PocketQuery**[127] is a database focused on potentially druggable sites for inhibition of PPIs with small molecules. All PDB entries containing PPIs are present in PocketQuery, and are annotated with a score estimating the ligandability of the PPI interface, energetic properties, structural properties, evolutionary properties, and a list of the relevant residues. This score is generated by a structure-based machine learning method trained on existing PPI inhibitors and their binding microenvironments [139]. The creators of PocketQuery note that the sites listed in the database are not binding hotspots nor the entire PPI interface, but rather clusters of residues at which small molecule drug design to inhibit the interaction can start.

#### 4.2.2 Databases predicting novel pockets: whole human proteome

**HProteome-Bsite**[120] contains proteome-wide binding site predictions, as well as predicted small molecule ligands for the predicted binding sites. Both types of predictions are made by the GalaxySite method [140], a docking-based method informed by sequence and structure templates. All protein structures in HProteome-Bsite are computationally predicted by AlphaFold2; no structures from the PDB are included.

**PrePCI**[129] is a database constructed by running a method (also called PrePCI) that predicts binding pockets and ligands for all human AlphaFold2 protein structures. Like HProteome-Bsite, predictions are only made for computationally predicted structures, although known binding interactions present in the PDB are listed alongside predictions. The PrePCI method is template-based, using both protein sequence and structure similarity to proteins with known pockets in the PDB to identify pockets on the query protein, and chemical fingerprint similarity of compounds to predict ligands.

**CaviDB**[114] is a database of fpocket [62] predictions for all structures in the PDB and the AlphaFold2 database available in 2022. The entire human proteome as contained in the AlphaFold2 database is therefore in CaviDB, in addition to proteomes of several other organisms of general scientific interest. As described in Section 3, fpocket is a commonly-used geometric method that detects cavities on the query protein surface. The existence of CaviDB obviates the need to recompute fpocket predictions for the included proteins, unless one wishes to use parameters different from the program default. In addition to pocket residues, CaviDB also provides various pocket-level and protein-level features. CaviDB does not include predicted ligands.

**CavitySpace**[115] is also a proteome-scale database of computed cavities. Pocket predictions are made with the CAVITY tool [59] which, like fpocket, is geometry-based. In addition to predicting binding pockets, CAVITY also provides a score of predicted druggability (called the CavityDrugScore) for each pocket. The CavityDrugScore is based on the number of hydrophobic grid points within a pocket and was trained and validated with a set of highly druggable and less druggable proteins; these proteins were selected to assess druggability in particular, not just ligandability [59]. CavitySpace presents all predicted pockets partitioned into three groups according to their CavityDrugScore: strong, medium, or weak. CavitySpace is focused on the human proteome, containing predictions for all human proteins in both the PDB and the AlphaFold2 database.

**PrankWeb**[128] uses the P2RANK method [89] to predict pockets. P2RANK is a machine learning method that operates on protein structure. PrankWeb contains pocket predictions for the entire PDB, the model organism proteomes contained in the AlphaFold2 database, and the Swiss-Prot component of the AlphaFold2 database. For each predicted pocket, PrankWeb also provides estimates of evolutionary conservation. If one wishes to compute pockets for a protein not included in the database, they may upload a PDB file for on-the-fly pocket prediction. PrankWeb does not include predicted ligands.

**ProBiS-Fold**[131] is the counterpart to the previously-described ProBis-Dock database [130]; whereas ProBis-Dock contains all instances of protein-ligand binding in the PDB, ProBis-Fold contains predictions of all binding sites in AlphaFold2 structures for the entire human proteome. Like the ProBis-Dock database, the predicted binding sites contained in ProBis-Fold were predicted by the structure-template-based ProBis algorithm [138]. The ProBis algorithm also provides predicted ligands or glycosylations for each site.

#### 4.2.3 Databases predicting novel pockets: known binding affinity values

**eModel-BDB**[117] is another database with predicted binding sites, though not across the entire PDB or the entire human proteome. The protein-ligand interactions in eModel-BDB are obtained from BindingDB (described in Section 5) and all have associated binding affinities. Protein complexes were modeled through a template-based approach with physics-based refinement. Binding pockets were predicted using eFindSite, a template-based pocket-finding algorithm [80]. The predictions were made using PDB structures deposited before February 2017, and validated using structures deposited to the PDB after February 2017.

### 4.3 Pocket databases for particular applications

#### 4.3.1 Pocket databases for particular applications: drug- and disease-relevance

**TTD (Therapeutic Target Database)**[135] is a database of potential human disease targets and multiple different aspects of their druggability, including known binding pockets. TTD goes beyond ligandability to capture druggability through three perspectives: molecular interactions, cell-based expression variations, and human system features. For each target entry, TTD provides all PDB structures of the target complexed with a ligand, with binding residues listed for each PDB structure. Target entries also include related diseases, drugs known to bind the target along with their approval or clinical trial status, similar human proteins, tissue distribution, and associated pathways. TTD also contains drug entries, which include properties of the drug, known targets, and known associated pathways.

**Pharos**[126] is an NIH resource that serves as a publicly-available interface for the Target Central Resource Database (TCRD), which was created to increase understanding of understudied protein families and broadly characterize their potential druggability. Pharos harmonizes data from 79 gene and protein databases. It is organized into sections for targets, diseases, and ligands, each of which allows the user to browse the respective entries. Target entries include expression data, approved drugs, known ligands, known and predicted PPIs, associated pathways, disease associations, and orthologs. Each target in Pharos is assigned a Target Development Level (TDL) based on its degree of prior knowledge as a drug target: Tclin (targets with approved drugs), Tchem (targets without approved drugs but with known small molecule binding activity), Tbio (targets without known small molecule binding activity but with functional annotations or presence in literature), or Tdark (targets without known binding activity or functional annotations but present in UniProt). Binding pocket information is included in target entries through sequence annotations. It is important to note that because Pharos includes many under-characterized targets by design, not all targets with associated ligands have annotated binding residues. Disease entries include associated targets, predicted targets, and parent and child nodes of the disease in the hierarchy of all included diseases. Each disease is characterized by how many of each of the four TDLs are associated with it. Ligand entries include basic compound information and links to similar compounds, known targets, and predicted targets.

**PDID (Protein-Drug Interaction Database)**[125] is a database of both known and predicted protein-drug interactions across all human proteins present in the PDB. Known interactions were obtained from DrugBank, BindingDB, and the PDB. Predicted interactions were obtained by running three different binding site prediction methods (ILbind [141], SMAP [142], and eFindSite [80]) on every pair of protein structures and drugs included in the database. The three prediction methods are a consensus-based machine learning method and two different template-based methods, respectively. Known and predicted binding sites are provided in the form of the drug's central coordinates with respect to the relevant PDB structure.

#### 4.3.2 Pocket databases for particular applications: particular types of ligands

**MetalPDB**[124] is a database of metal binding sites on proteins in the PDB. MetalPDB both includes metal binding sites that are present in PDB structures and potential metal binding sites predicted by a conservation-based approach. Each entry in MetalPDB represents a metal-binding site on a PDB structure and includes the residues in the binding site, coordination geometry, endogenous and exogenous ligands, and any equivalent or equistructural sites across different structures. Users can search the database contents on the website by protein or by metal. MetalPDB contains sites for over 60 different metals.

**MbPA (the Metal-binding Protein Atlas)**[123] is also a database of binding sites on metalloproteins. Unlike MetalPDB, MbPA contains proteins that do not have an experimentally-solved structure; proteins predicted to bind metals that have sequences in UniProt but no structures in the PDB are modeled by AlphaFold2. MbPA includes proteins with metal binding sites observed in PDB structures, proteins with metal binding sites inferred through experiments other than structure determination, and proteins with metal binding sites inferred computationally (*e.g.* through functional analysis, sequence templates, or homology modeling). Each protein entry in MbPA includes relevant PDB and/or AlphaFold2 structures, metal binding sites in the sequence context, and functional annotation with GO codes. MbPA also includes information about relationships between metal binding sites and disease, with an annotated list of pathogenic mutations in metal binding sites. While MbPA includes more proteins than MetalPDB by virtue of its use of computationally-predicted structures, it does not include as many metal types as MetalPDB.

**ZincBind**[137] is a database exclusively dedicated to zinc binding sites found in the PDB. The data was assembled by extracting all PDB structures containing zinc, clustered at 90% sequence identity to combat redundancy. ZincBind distinguishes between structures in which zinc is part of a binding site and structures in which zinc is only present as part of a salt. Each entry contains basic PDB structure information and the residues in each zinc binding site. The authors differentiate ZincBind from MetalPDB through its derivation of zinc binding sites through biologically relevant assemblies rather than from asymmetric units. Additionally, the ZincBind website contains a machine learning tool to predict zinc binding sites *de novo* from sequence or structure [143].

**DeepCholesterol**[116] is a database of cholesterol binding sites on membrane proteins predicted by docking. Docking experiments were only performed on experimentally-determined protein structures. The data is divided into deep versus interfacial cholesterol binding sites, and then further organized by protein family. Each protein is either designated as not predicted to have a cholesterol binding site, or is annotated with a list of binding residues and predicted binding energy. Deep binding sites also contain donor and acceptor information.

**PROCARB**[132] is a database of carbohydrate binding sites. The main component of PROCARB is a set of PDB structures complexed with carbohydrates. PROCARB also contains homology models of glycoproteins with no experimentally determined structure (as of PROCARB's release) with glycosylation sites predicted by the 3D-JIGSAW [144] method, which performs homology modeling. The browse and search functionalities of the PROCARB website are currently broken, but one can still download the full dataset.

#### 4.3.3 Pocket databases for particular applications: particular types of targets

**KLIFS (Kinase-Ligand Interaction Fingerprints and Structures)**[122] is a database dedicated to structural information related to kinase interactions, motivated by the key role of kinases in signal transduction and as drug targets. KLIFS contains human and mouse kinases present in the PDB, with each kinase entry containing general PDB structure information, kinase-specific information (*e.g.* angles and rotations of key kinase motifs), waters and residues at the binding site, any known binding affinities, and which of the main pockets and subpockets present in kinases the ligand targets. KLIFS also lists kinase-targeting drugs. Each drug is linked to any structure entries that have the drug complexed with a kinase, any structure entries that have an analog of the drug complexed with a kinase, and any bioactivity data. Users can search the KLIFS website by kinase classification, desired interaction location or targeted subpocket, or kinase-specific structural conformation.

#### 4.3.4 Pocket databases for particular applications: particular modes of binding

**AroCageDB**[110] is a database of binding pockets with the aromatic cage structural motif. Aromatic cages are important for molecular recognition and many proteins with the aromatic cage motif have been shown to be therapeutically relevant. AroCageDB includes all PDB structures with a ligand bound to aromatic residues in an orientation consistent with the definition of an aromatic cage. Each included structure entry includes basic PDB information, pocket properties, basic ligand information, and any available binding affinity values. Users of the AroCageDB website can visually inspect complex visualizations to note binding residues, which are shown interacting with the ligand, colored, and labeled. Explicit lists of pocket residues are available for download.

**ASD (AlloSteric Database)**[111] is a database focused on allosteric binding. Allosteric binding refers to when a ligand binds at a site other than the primary functional site. Designing drugs to target an allosteric binding site has rendered previously “undruggable” targets ligandable. ASD contains both known allosteric sites, obtained from PDB and the literature at large, as well as allosteric sites predicted by AllositePro [145]. ASD contains both protein and ligand (termed “modulator”) entries. Each protein entry contains functional annotations, any available PDB structures, binding residues of its allosteric sites, allosteric mechanisms, and a list of ligands. Each ligand entry includes basic ligand properties, targets (along with whether the ligand activates, inhibits, or regulates the target), and diseases associated with its targets. Additionally, the ASD website contains numerous allostery-related features and tools, such as the Allo-PPI dataset of allosteric PPI modulators, the Allo-Mutation dataset of mutations in allosteric sites with cancer pathogenicity, and the AlloScore tool for predicting allosteric protein-ligand binding affinities.

### 4.4 General knowledge bases with pocket information

**UniProt**[136] is a central knowledge base of protein sequences that is widely used – including by many databases listed in this review – to obtain reference proteomes for a vast array of species. UniProt consists of UniRef (protein sequence clusters are various sequence identity percentages), UniParc (non-redundant archive of protein sequences), and UniProtKB (the UniProt knowledge base). UniProtKB consists of Swiss-Prot, in which all sequences are manually reviewed, and TrEMBL, in which sequences are unreviewed. If a protein in Swiss-Prot is known to have a binding site, the residues in its binding site are listed in the Function section.

**InterPro**[121] is a protein knowledge base focused on protein family classification and functional analysis. It synthesizes annotations from many other databases, such as Pfam, PROSITE, and SUPERFAMILY. While not perfectly overlapping with UniProtKB, InterPro covers most of UniProt as of 2022. Binding sites are one of the many types of functional annotations included in InterPro, and are represented as sequence motifs. InterPro includes both known binding sites and predicted binding sites. Predicted binding sites were obtained by InterProScan, a hidden Markov model that uses similarity to known protein signatures to infer functional annotations.

**SIFTS (Structure Integration with Function, Taxonomy and Sequences)**[134] is a database that connects UniProt with the PDB (specifically PDBe, the European PDB). One of the goals of SIFTS is to harmonize protein sequence annotations and protein structural annotations contained in these databases as well as other resources such as Gene Ontology, InterPro, Pfam, CATH, and SCOP. All of the binding pocket information contained in these resources is therefore also present in SIFTS. The SIFTS website does not contain a browsable version of the data; users must access the data through Application Programming Interface (API) or flat file download.

**The G2P portal (Genomics 2 Proteins portal)**[119] is similar to SIFTS in that it is a database linking protein sequence and structure information. G2P is distinct from SIFTS in that it includes millions of genetic variants, with the portal's primary purpose being to map sequence and structure information to genetic variation at the proteome scale. Genetic variant information comes from ClinVar [146], HGMD [147], and gnomAD [148]; sequence annotations come from UniProt; and structure information comes from the PDB and AlphaFold Protein Structure Database. Pocket information is included as active site residues as annotated in UniProt, when present. One way in which users can use the database is by mapping variants in the sequence and structure space and noting overlap with known binding sites. While individual protein entries are downloadable as TSVs, there is no way to download the entirety of the database together.

We emphasize that these four databases contain a wealth of information beyond binding pockets; their inclusion as pocket databases in this review is meant to reflect the fact that they have binding pocket information, not that their focus is on binding pockets.

## 5 Interaction databases

There exist many databases that do not contain information on specific residues or coordinates that make up the binding pocket, but do contain information on known or predicted protein-ligand binding interactions. We define these as “interaction databases.” Note that, under our definitions, not all pocket databases are interaction databases; a pocket database based on cavity prediction that does not contain known or predicted ligands associated with its pockets would not be an interaction database. Similarly, not all interaction databases are pocket databases; an interaction database with literature-derived data on protein-ligand interactions that does not have corresponding complexed structures and no residue interaction information would not be a pocket database. We identified a total of 63 interaction databases (Table S2), 42 of which have a website or download currently available (Table 2).

**Figure:**
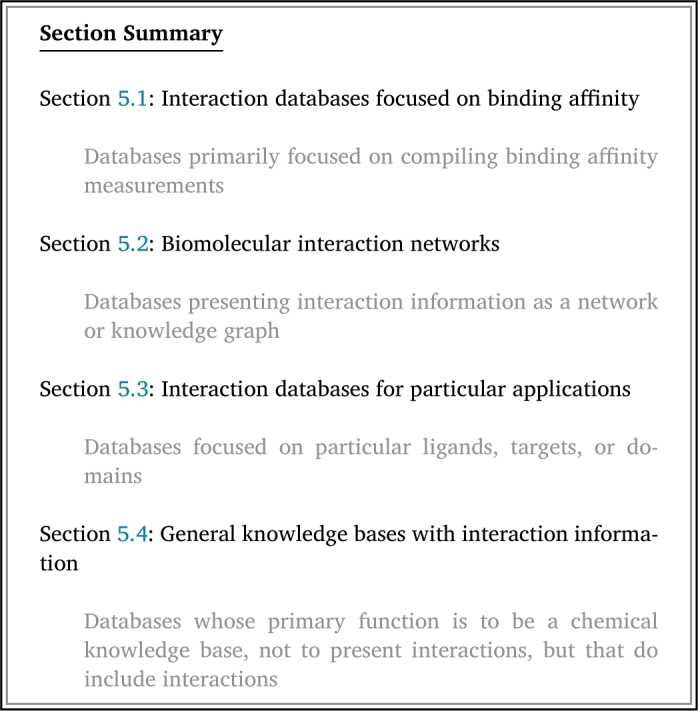

**Table 2:** Details for all interaction databases described in this review, along with most recent reference and active URL as of January 2024. Labels correspond with subsection in which databases are described in the body of the review. The “pocket database” label indicates that the database can also be found in Table 1 and is described in the Pocket Databases section of the main text. A “browsable” website refers to a website on which the user can explore the contents of the database without downloading files or using an Application Programming Interface (API). A database is denoted with “download available” if the entirety of the database is available to download at once. See Table S2 for more information.

Name	Year Created	Last Updated	Labels	Browsable Website Available	Download Available	API Available	URL
AroCageDB [110]	2021	2021	Pocket database	yes	yes	no	https://drug-discovery.vm.uni-freiburg.de/arocagedb/
ASD [111]	2011	2023	Pocket database	yes	yes	no	https://mdl.shsmu.edu.cn/ASD/
BIND [149]	2000	2006	Interaction network	no	yes	no	http://bind.ca/
Binding MOAD [112]	2005	2023	Pocket database	yes	yes	no	http://www.bindingmoad.org/
BindingDB [150]	2001	2024	Binding affinity	yes	yes	yes	https://www.bindingdb.org/rwd/bind/index.jsp
BioLiP [34]	2013	2024	Pocket database	yes	yes	yes	https://seq2fun.dcmb.med.umich.edu//BioLiP/index.cgi
CASF [113]	2007	2016	Pocket database	no	yes	no	http://www.pdbbind.org.cn/casf.php
ChEMBL [151]	2009	2023	Compound knowledge base	yes	yes	yes	https://www.ebi.ac.uk/chembl/
dbHDPLS [152]	2018	2018	For particular applications;	no	yes	no	https://www.dlearningapp.com/web/dbDPLS/index.php
			Drug- and disease-relevance				
DeepCholesterol [116]	2018	2018	Pocket database	yes	no	no	https://deepcholesterol.soton.ac.uk/
DrugBank [153]	2006	2024	Compound knowledge base	yes	yes	yes	https://go.drugbank.com/
DTC [154]	2017	2019	Binding affinity	yes	yes	yes	https://drugtargetcommons.fimm.fi/
eModel-BDB [117]	2018	2018	Pocket database	yes	yes	no	https://brylinski.org/emodel-bdb-0
FireDB [118]	2007	2020	Pocket database	yes	yes	yes	https://firedb.bioinfo.cnio.es/Php/FireDB.php
GtoPdb [155]	2014	2023	For particular applications;	yes	yes	yes	https://www.guidetopharmacology.org/
			Drug- and disease-relevance				
Het-PDB Navi [107]	2004	2024	Pocket database	yes	no	no	https://hetpdbnavi.nagahama-i-bio.ac.jp/
HProteome-BSite [120]	2023	2023	Pocket database	yes	yes	no	https://galaxy.seoklab.org/hproteome-bsite/database/
IntAct [156]	2004	2024	Interaction network	yes	yes	yes	https://www.ebi.ac.uk/intact/home
KLIFS [122]	2014	2024	Pocket database	yes	yes	yes	https://klifs.net/
LigBase [108]	2002	2003	Pocket database	yes	no	no	https://modbase.compbio.ucsf.edu/ligbase/
MbPA [123]	2023	2023	Pocket database	yes	yes	yes	http://bioinfor.imu.edu.cn/mbpa/
MeDBA [157]	2020	2022	For particular applications;	yes	no	no	https://medba.ddtmlab.org/
			Particular ligands				
MetalPDB [124]	2013	2023	Pocket database	yes	yes	yes	https://metalpdb.cerm.unifi.it/
MIPS [158]	2009	2024	For particular applications;	yes	no	no	http://dicsoft2.physics.iisc.ac.in/mips/
			Particular ligands				
PDBbind [159]	2004	2024	Binding affinity	yes	yes	no	http://www.pdbbind.org.cn/
							https://www.pdbbind-plus.org.cn/
PDBSite [103]	2000	2014	Pocket database	yes	no	no	http://wwwmgs.bionet.nsc.ru/mgs/gnw/pdbsite/
PDBsum [106]	1997	2023	Pocket database	yes	yes	no	https://www.ebi.ac.uk/thornton-srv/databases/pdbsum/
PDID [125]	2014	2015	Pocket database	yes	yes	no	http://biomine.cs.vcu.edu/servers/PDID/index.php
PDSP Ki [160]	1999	2021	Binding affinity	yes	yes	no	https://pdsp.unc.edu/databases/kidb.php
Pharos [126]	2016	2023	Pocket database	yes	yes	yes	https://pharos.nih.gov/
PLBD [161]	2021	2024	Binding affinity	yes	yes	no	https://plbd.org/db/
PrePCI [129]	2023	2023	Pocket database	yes	no	no	https://honiglab.c2b2.columbia.edu/prepci.html
ProBiS-Dock [130]	2021	2021	Pocket database	yes	yes	no	http://probis-dock-database.insilab.org/
ProBiS-Fold [131]	2022	2022	Pocket database	yes	yes	no	http://probis-fold.insilab.org/
PROCARB [132]	2008	2016	Pocket database	no	yes	no	https://www.procarb.org/procarbdb/
PROMISCUOUS [162]	2011	2020	For particular applications;	yes	yes	no	https://bioinf-applied.charite.de/promiscuous2/index.php
			Drug- and disease-relevance				
PSCDB [163]	2011	2012	For particular applications;	yes	yes	no	https://togodb.biosciencedbc.jp/togodb/view/pscdb
			Particular mode of binding				
Q-BioLiP [133]	2023	2024	Pocket database	yes	yes	no	https://yanglab.qd.sdu.edu.cn/Q-BioLiP/
scPDB [102]	2004	2017	Pocket database	yes	yes	no	http://bioinfo-pharma.u-strasbg.fr/scPDB/
STITCH [164]	2008	2016	Interaction network	yes	yes	yes	http://stitch.embl.de/
TTD [135]	2002	2024	Pocket database	yes	yes	no	https://db.idrblab.net/ttd/
ZincBind [137]	2019	2022	Pocket database	yes	no	yes	https://zincbind.net/

Twenty-six of the available interaction databases are also pocket databases, and have been described in the previous section. These are AroCageDB, ASD, Binding MOAD, BioLiP, CASF, DeepCholesterol, eModel-BDB, FireDB, Het-PDB Navi, HProteome-BSite, KLIFS, LigBase, MbPA, MetalPDB, PDBSite, PDBsum, PDID, Pharos, PrePCI, ProBiS-Dock, ProBiS-Fold, PROCARB, Q-BioLiP, scPDB, TTD, and ZincBind.

### 5.1 Interaction databases focused on binding affinity

**BindingDB**[150] was the first binding affinity database established. Rather than reporting a binary relationship of interaction or non-interaction, BindingDB and all other binding affinity databases listed here provide experimentally-determined affinity measurements for every included entry. The affinity data in BindingDB is manually and automatically curated from the scientific literature, patents, other databases, and direct deposition by experimentalists.

**PDBbind**[159] contains affinity measurements for all protein-ligand interactions present in the PDB. PDBbind is split into three sets of increasing quality: the general set, the refined set, and the core set. As of early 2024, PDBbind is in the midst of a transition to a new website and access model. The original website (www.pdbbind.org.cn) will remain available but will not be updated past PDBbind version 2020. The new website (www.pdbbind-plus.org.cn; denoted as PDBbind+) contains PDBbind version 2021 and will host subsequent releases. PDBbind+ has introduced a subscription-based model, in which paying subscribers can access more recent and higher volume data and unlimited access to additional tools and services; no other database included in this review is paywalled.

**DTC (Drug Target Commons)**[154] is a community-driven (as opposed to being curated by one central group) repository of annotated drug-target bioactivity data. DTC harmonizes heterogeneous bioactivity assays through their micro bioassay ontology (*μ*BAO). Its creators propose that this new ontology, along with decentralized annotation, allows for larger-scale annotation across assay types without conflating results of different assays.

**PDSP Ki (Psychoactive Drug Screening Program Ki)**[160] is a database of inhibition constants (Ki) for psychoactive drugs. It focuses on drugs that target G-protein coupled receptors, ion channels, and transporters.

**PLBD (Protein-Ligand Binding Database)**[161] is a recently-created database that contains multiple different thermodynamic and kinetic properties for protein-ligand interactions and is currently focused on human carbonic anhydrases and heat shock proteins. PLBD distinguishes itself from existing binding affinity databases not only by containing parameters such as change in enthalpy and entropy, but also by accounting for protonation effects to better compare data across different experiments.

### 5.2 Biomolecular interaction networks

**BIND (the Biomolecular Interaction Network Database)**[149] is a curated database of interactions between proteins, nucleic acids, and small molecules. Protein-protein interactions dominate the database; the next most common interactions are protein-DNA interactions and protein-small molecule interactions. While the BIND website has been taken down and the resource is no longer actively maintained, a download of the database's final release from 2006 is available [165].

**IntAct**[156] is another database of biomolecular interactions. Like BIND, IntAct is primarily made up of PPIs, but also contains interactions between proteins and small molecules. The IntAct authors note that, unlike in the field of protein structure, there has not been one large central database for molecular interactions; instead, several small database efforts arose independently [156]. This phenomenon explains the simultaneous existence of BIND and IntAct. In 2013, IntAct merged with another similar interaction network dataset, MINT [166]. In addition to downloads and API capabilities, the IntAct website provides a network interface, allowing the user to visualize the interaction relationships for a protein or compound of interest.

**STITCH (Search Tool for Interacting Chemicals)**[164] is a database focused on interactions between proteins and small molecules, rather than primarily on PPIs – though PPIs are included. Like IntAct, STITCH provides a network visualization tool on its website. The user can filter this network for species- or tissue-specificity. BIND, STITCH, and IntAct are all curated from scientific literature.

### 5.3 Interaction databases for particular applications

#### 5.3.1 Interaction databases for particular applications: drug- and disease-relevance

**GtoPdb (the IUPHAR/BPS Guide to PHARMACOLOGY)**[155] is a database of drugs and drug targets. Target entries include general gene and protein information, tissue distribution, clinically-relevant mutations, agonists, antagonists, and allosteric modulators. Binding affinity is provided with ligands when available. Drug entries include general information, bioassay data, clinical data, and similar ligands. GtoPdb is curated by a team of experts which collaborates with NC-IUPHAR subcommittees. In addition to the general GtoPdb, its creators have also established GtoImmuPdb (Guide to IMMUNOPHARMACOLOGY) and GtoMPdb (Guide to MALARIA PHARMACOLOGY), which are portals to immunology-related and malaria-related entries of GtoPdb, respectively. They also biannually publish the Concise Guide to PHARMACOLOGY, which provides an overview of the online GtoPdb database at a snapshot in time.

**PROMISCUOUS**[162] is a network database of PPIs and target-drug interactions established for use in drug repurposing and polypharmacology. Like the previously-described biomolecular interaction network databases, PROMISCUOUS can be viewed as a knowledge graph and includes network visualization on its website; but unlike the previously-described network databases, PROMISCUOUS only includes drugs, drug-like compounds, and drug targets as nodes in the knowledge graph. Users can search the network by compound or target, and can find potential indications for their drug of interest or potential drugs for their indication of interest as predicted by machine learning methods and structural similarity. Data in PROMISCUOUS is collected from the chemical knowledge bases ChEMBL and DrugBank (described in Section 5.4), and the now-unavailable SuperDrug2 [167] and SuperTarget [168]. PROMISCUOUS does not include binding affinity information, but does include adverse drug reactions.

**dbHDPLS (Database of Human Disease Protein-Ligand Structure)**[152] is a database containing complexed structures, binding affinities, and general information related to human disease. The database is constructed by taking all disease-associated human protein-ligand structures from the PDB and annotating them with information from DrugBank, UniProt, BioLiP, PDBbind, and Binding MOAD. At the time of writing, the dbHDPLS website does not allow for browsing or searching of its data entries, but the raw dataset in spreadsheet form and a Cytoscape [169] visualization of the drug-target binding network are available for download.

#### 5.3.2 Interaction databases for particular applications: particular types of ligands

**MIPS (Metal Interactions in Protein Structures)**[158] is a database of all metalloproteins (proteins containing metal ions) structures in the PDB. On the website interface, users can search for structures by metal ion or by types of metal ion interaction. Users can also view the residues that are within a specified distance cutoff of a given metal in a MIPS structure entry.

**MeDBA (Metalloenzyme Data Bank and Analysis platform)**[157] is a database dedicated to comprehensive information on metalloenzymes, which are a subclass of metalloproteins in which the bound metal plays a role in catalytic activity. MeDBA categorizes metalloenzymes into three types according to if the metal cofactor is present in the active site and if so, how tightly it is bound. Each metalloenzyme is annotated with its metal cofactors, catalytic activity, associated ligands, and structures if experimentally determined. When a PDB structure is available, MeDBA provides the metal binding pharmacophore and the metal chelating residues.

#### 5.3.3 Interaction databases for particular applications: particular modes of binding

**PSCDB (Protein Structural Change Database)**[163] is a database of proteins which undergo large structural change upon ligand binding. Each entry in the database includes a pair of PDB structures – one in which the ligand is bound, and one without a ligand. Entries also describe the type of motion and how it relates to the act of ligand binding, the fixed and moving segments of the protein, and the RMSD between the bound and unbound forms.

### 5.4 General knowledge bases with interaction information

**ChEMBL**[151] is a database of drug-like bioactive molecules. It is manually curated, with bioactivity data extracted from peer-reviewed scientific literature. Each compound in ChEMBL is represented by a “Compound Report Card,” which includes compound representations, literature references, and calculated properties. Protein interactions for each compound are presented as mechanisms, metabolism pathways, bioactivity and assay data, and predicted targets.

**DrugBank**[153] is a database of FDA-approved drugs and drugs seeking FDA approval. Like ChEMBL, DrugBank is manually curated. Whereas ChEMBL focuses on bioassay data, DrugBank contains more information on clinical trials and drug products. Protein interactions for each drug are presented as mechanisms of action and metabolic pathways, with no known or predicted off-targets present.

As similarly noted in Section 4.4, these more general knowledge bases contain a wealth of information beyond protein-ligand interactions, and are included here to reflect that they contain this interaction information among other chemical knowledge.

## 6 How are these databases used?

The majority of the databases (47/53) described in this review are present on browsable websites. While requiring more work to deploy and maintain than uploading a compressed version of the data to a repository hosting platforms such as Zenodo or SimTK, the ability to quickly browse the contents of the database without needing to download a large file or to write a single line of code reduces the barrier to using the dataset. Beyond exploring and familiarizing oneself with the data, one can also perform small-scale analyses. One example of small-scale database usage is the use of KLIFS to check the binding pocket of the p21-activated Kinase 4 when designing a selective inhibitor [170]. DeepCholesterol was used to identify cholesterol binding sites in zebrafish cystic fibrosis transmembrane conductance regulator (CFTR) for comparison to human CFTR [171]. MetalPDB was used to infer metal-binding constraints to model newly-discovered zinc binding sites of human ADAR1 [172]. TTD was used to check if there were previously known interactions between a compound of interest and a set of predicted targets [173]. We note that it is possible that the work in these particular examples was done using a downloaded version of the database or an API; we cannot decisively state that these were all conducted using just the browsable website. However, the point stands that all of these analyses are possible using only the browsable website, illustrating that such websites enable scientists to obtain knowledge relevant to their particular applications quickly, easily, and without requiring computational expertise.

That being said, manually obtaining information from a browsable website is often not sufficient for larger-scale analyses, which are more easily conducted with a downloaded copy of the dataset or the use of an API. A flat file download allows the user to access all data simultaneously and use data science techniques to conduct complex analyses if desired. Using an API requires comfort with writing code, making it less accessible than a browsable website or flat file download, but enables automated large-scale analysis without needing the memory space to download an entire database. One example of such large-scale analyses is how Kibble et al. used TTD to characterize known targets of all FDA-approved drugs for comparison with those of natural products [174]. Sorgenfrei et al. and Jaeger et al. used KLIFS to identify binding pockets for 305 and 284 kinases, respectively, to compute features in their datasets for training and testing ML models for screening small molecules for kinase inhibition activity [175], [176]. Saldaño et al. used BioLiP to create a dataset of 91 bound and unbound protein conformations to evaluate if AlphaFold2 preferentially predicts one conformation over the other [177]. Multiple groups have conducted virtual screens of thousands of targets from sc-PDB [178], [179], [180]. While technically feasible with online browsing and individual entry downloads, these tasks become realistically manageable with a downloaded dataset or API. 40/53 of the databases described in this review have a download available, and 19/53 have an API.

The past decade has seen an explosion of machine learning for biology [181]. ML models, particularly deep neural networks, require large training datasets. Many pocket databases and interaction databases have been used as training and testing data for ML. One example is the work of Coelho et al., in which a dataset was created from the combination of DrugBank, BindingDB, and BioLiP to train an ML model to predict protein-ligand interaction [182]. Similarly, PDBbind and CASF are frequently used as datasets to train, validate, and test ML models for predicting protein-ligand binding affinity [183], [184], [185] and ligand binding pose prediction [186], [187]. CASF has also been used as an external test set for a 3D linker design generative model [188]. Miljković et al. created a dataset of kinase inhibitors with diverse binding modes for an ML classifier using KLIFS [189]. BioLiP is frequently used to create training and testing datasets for machine learning models that aim to predict small molecule binding pockets [78], [190], [191], nucleic acid binding pockets [192], PPI sites [193], [194], and general protein function prediction at the residue-level [195], [196]. Sang et al. used TTD to construct training and testing sets for their drug-disease association prediction model [197]. Protein-ligand interaction data from STITCH has been used to train ML models for predicting drug-target interactions [198], adverse drug reactions [199], and drug-drug interactions [200], [201]. We emphasize that this is a non-exhaustive list of previous use of the described databases for ML models focused on tasks related to virtual screening and drug development, and that in the coming years the amount of such research will only continue to increase.

## 7 Comparison of database contents

With dozens of pocket databases currently available, it is expected that there will be both redundancies and discrepancies in the pockets provided for a given protein present in multiple databases. To illustrate this, we provide three human proteins and one viral protein as case studies: purine nucleoside phosphorylase (PNP) (PDB ID: 1YRY), the CNOT6L nuclease domain (PDB ID: 3NGQ), Mitogen-activated protein kinase kinase kinase 14 (MAP3K14) (PDB ID: 4DN5), and the SARS-CoV-2 spike protein (PDB ID: 7L4Z). For each protein, we present their pockets as reported by six of the databases described in this review: three databases with known pockets extracted from the PDB (BioLiP, MbPA, and sc-PDB), two databases of predicted pockets (CASTp and CaviDB), and one general knowledge base with binding annotations (UniProt).

We selected these four proteins in particular to serve as illustrative examples due to their biological and clinical significance. PNP is part of the purine salvage pathway. It is associated with immunodeficiency and has been proposed as a drug target for immunodeficiency-related conditions, with one approved drug [202], [203], [204]. CNOT6L is part of a deadenylase complex that has been proposed as a drug target for obesity [205], [206]. We selected this protein as an example of a potential drug target without existing approved drugs, as well as an example of a structure with metal ion cofactors. Kinases are key drug targets due to their importance in many signaling pathways [122]; we selected MAP3K14 as an example of a kinase. The SARS-CoV-2 spike protein mediates viral entry and its mutations have resulted in novel variants of COVID-19, driving the ongoing global pandemic [207], [208], [209], [210]. We selected this protein as our fourth case study due to the self-explanatory high levels of interest in it for drug and vaccine development campaigns.

### 7.1 Case study I: purine nucleoside phosphorylase (1YRY)

**Fig. 2:**
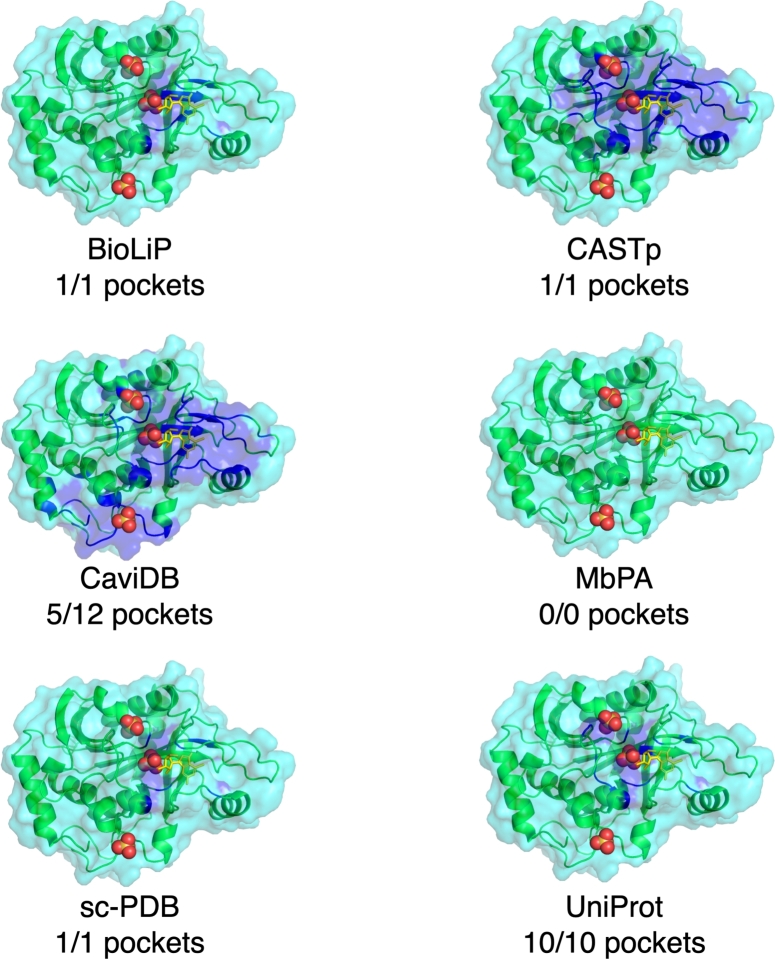
Purine nucleoside phosphorylase (green ribbons, cyan surface) complexed with 7-methyl-6-thio-guanosine (yellow sticks) and sulfate ions (red and yellow spheres) (PDB ID: 1YRY). Pockets from the protein entry in the six databases shown with blue ribbons and surface. Up to the top five pockets, or all UniProt sites, shown together.

The PNP structure was included in five of the six selected databases (Fig. 2); it was not present in MbPA as it is not a metal-binding protein. Visual inspection reveals that the five databases contain very similar pockets (shown with blue highlights in the figure), all highlighting the cleft in which the ligand (shown with yellow sticks in the figure) binds. The two databases which contain predicted pockets highlight larger pocket regions than the three that report previously known pockets (*i.e.* their blue highlighted pocket region is larger). This is especially apparent with CaviDB, which reported a total of 12 predicted pockets, of which we have only visualized five for ease of interpretation. Both CaviDB and CASTp recover the main cleft but also highlight the surrounding regions, reducing specificity. Closer inspection reveals slight differences between the extracted pockets from BioLiP, sc-PDB, and UniProt, but these are minor and do not change the end result of identifying the binding cleft.

### 7.2 Case study II: CNOT6L nuclease domain (3NGQ)

**Fig. 3:**
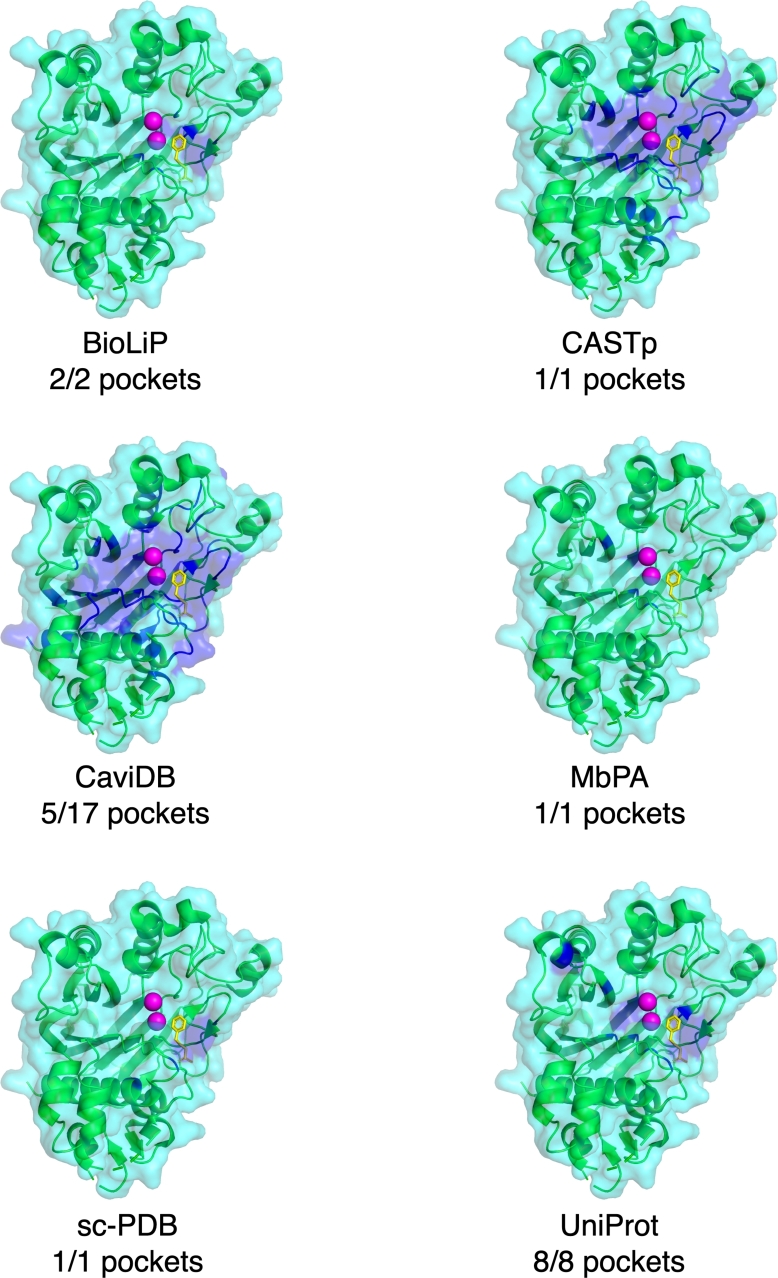
CNOT6L nuclease domain (green ribbons, cyan surface) complexed with 3-pyridinium-1-ylpropane-1-sulfonate (yellow sticks) and magnesium ions (magenta spheres) (PDB ID: 3NGQ). Pockets from the protein entry in the six databases shown with blue ribbons and surface. Up to the top five pockets, or all UniProt sites, shown together.

The nuclease domain was included in all six of the selected databases (Fig. 3). While all six databases highlighted pockets (shown with blue highlights in the figure) in the region around the bound ligand (shown in yellow in the figure), there is more variation between the six than in the previous case study. Unlike the previous case study, the databases that contain pockets extracted from the PDB have key differences. Whereas the sc-PDB pockets highlight an area around the ligand, the MbPA pocket highlights an area around the magnesium cofactors (shown in magenta in the figure); BioLiP highlights both. This is not surprising given the different purposes of these databases. The UniProt pockets reflect both the ligand and cofactor sites being annotated, in addition to a binding site far outside of the main cleft. As before, the predicted pockets are much larger than the extracted pockets. While both do recover the main cleft, CaviDB in particular highlights several other regions of the protein.

### 7.3 Case study III: mitogen-activated protein kinase kinase kinase 14 (4DN5)

**Fig. 4:**
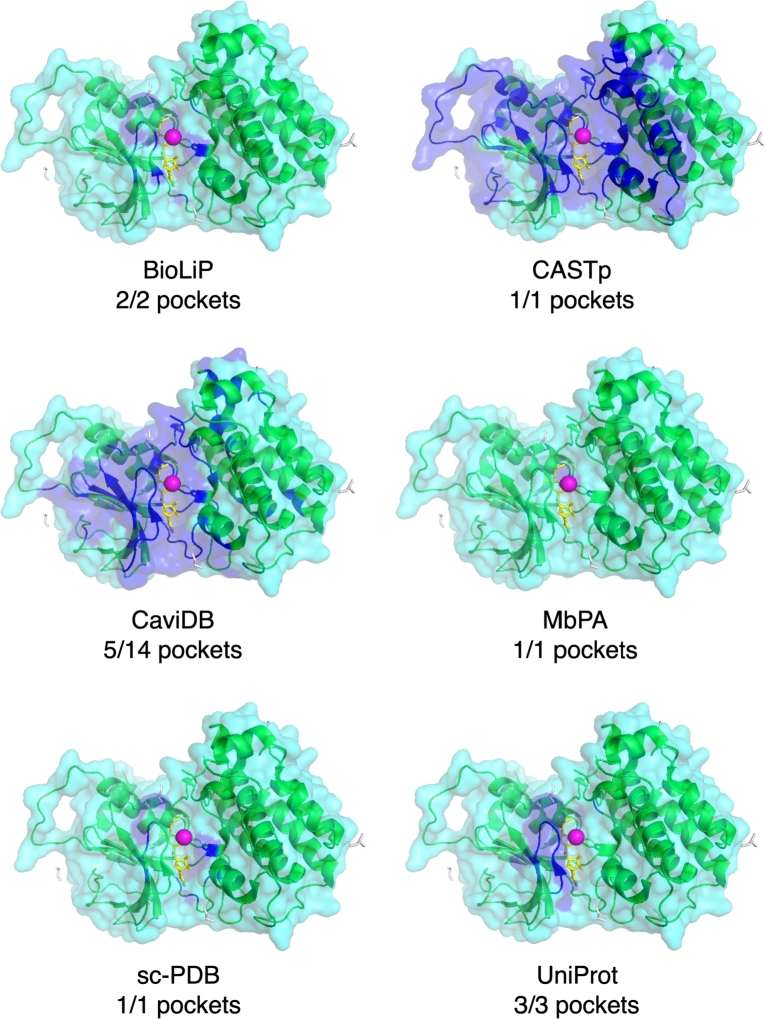
MAP3K14 (green ribbons, cyan surface) complexed with phosphothiophosphoric acid-adenylate ester (yellow sticks), glycerol (white sticks), 1,2-ethanediol (white sticks), and magnesium ions (magenta spheres) (PDB ID: 4DN5). Pockets from the protein entry in the six databases shown with blue ribbons and surface. Up to the top five pockets shown together. The original PDB file contains two identical chains with identical ligands; we only show chain A to avoid redundancy.

MAP3K14, also known as Nuclear Factor Kappa B-inducing kinase, was also present in all six of the selected databases (Fig. 4). The size and number of pockets (shown with blue highlights in the figure) varied across databases. As with the prior examples, the databases with known binding pocket annotations included fewer residues per pocket than the databases with predicted pockets. The pockets presented in the four databases of known pocket annotations varied based on whether the database focused on the metal cofactor, the ligand, or both.

### 7.4 Case study IV: spike protein (7L4Z)

**Fig. 5:**
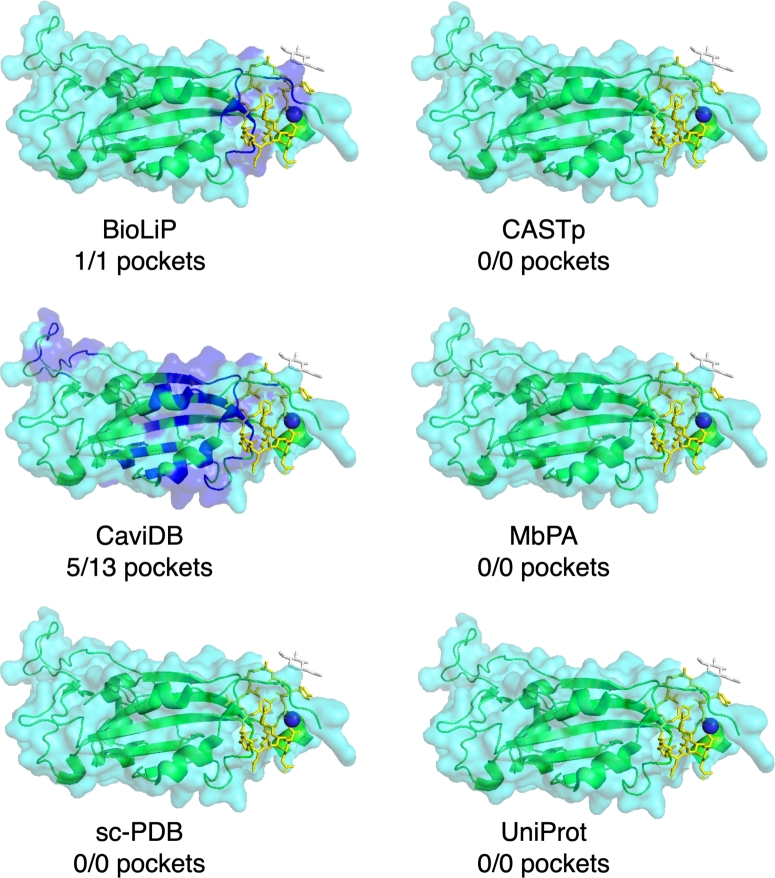
SARS-CoV-2 spike protein receptor binding domain (green ribbons, cyan surface) complexed with cyclic peptide (yellow sticks) and 2-acetamido-2-deoxy-beta-D-glucopyranose (white sticks) (PDB ID: 7L4Z). Pockets from the protein entry in the six databases shown with blue ribbons and surface. Up to the top five pockets shown together. The original PDB file contains multiple identical chains with identical ligands; we only show chain A to avoid redundancy.

We include the SARS-CoV-2 spike protein receptor binding domain due to its relevance to the ongoing pandemic and current drug discovery campaigns. Despite the scientific community having high interest in it, this structure had pockets listed in only two of the six selected databases (Fig. 5). sc-PDB and CASTp do not have this structure because they have not been updated since 2017 and 2018, respectively. MbPA does not have this structure because it is not a metalloprotein. UniProt contains an entry for the spike protein which includes this PDB structure, but the only annotated sites are cleavage sites, not binding sites, and the only annotated binding motifs are located outside the receptor binding domain. As in the other examples, the predicted pockets (CaviDB) are larger (*i.e.* the blue highlighted region is larger) and are located near and outside of the main known binding site (BioLiP).

## 8 Discussion

### 8.1 Accessibility

A theme that emerged in the compilation of Table 1, Table 2 was how sustained access to a database cannot be taken for granted. While we identified 79 databases as either pocket databases or interaction databases, 26 of those were completely unavailable, with no functional website, download, or API at the time of writing (Table S2). These databases contained valuable curated information, and while in some cases their discontinuation was motivated by redundancy with a database still available today, in many cases that information is now lost and would require spending more time and resources to recreate. We therefore encourage future database purveyors to carefully consider the longevity of their work; while a publication describing a database will outlast a defunct website, it does little good to the scientific enterprise to know that a dataset once existed but to be unable to access it.

There are multiple reasons why a database may become defunct. Websites cost money to host and require regular maintenance, so a loss of funding or departure of a webmaster is likely to lead to the website breaking and the database becoming inaccessible to the public. The creators of the database may also choose to take it down if it has become redundant with another database, if it is no longer realistically usable, or they foresee that a change in financial support will preclude its continued maintenance. Real examples of lost databases illustrate how common these scenarios are. Binding MOAD will become defunct in July 2024 despite its popularity due to added features significantly slowing the time to load each page [112]. The original BIND website was taken down in 2006 due to a loss of funding, prompting discussion in the community about the role of governmental funding for such databases [211], [212]. While the publicly-accessible, browsable website is gone, the BIND data is once again available as a static download [165] – highlighting the advantages of flat file download options with respect to sustained access.

### 8.2 Redundancy

Another theme that emerged was the similarity of many pocket and interaction database curation efforts. Unlike the area of protein structure determination, in which the PDB emerged as the one central database into which all newly-solved structures would be deposited, pocket and interaction databases have emerged independently across many different groups. This resulted in the existence of many similar, though not identical, datasets – *e.g.* eleven databases that contain known binding interactions extracted from the PDB but with slightly different features and criteria for which interactions to include. While our four case studies comparing the database contents largely showed coherence (with exceptions related to ligands versus cofactors, larger sizes of predicted pockets, and many cases where a database lacked a given structure), they also raise the question of why all these databases are necessary if many of them provide very similar information. With so many different versions of pocket data available, and especially different modes of predicting binding pockets across the human proteome, a tool to harmonize disparate pocket information is needed. This would be a non-trivial task, given the different pocket definitions used by these different databases and the different forms in which their data is accessible. However, once accomplished, a unifying resource made up of each individual database's contributions could leverage proteome-wide knowledge for downstream tasks such as virtual screening, drug repurposing, adverse drug reaction prediction, and more.

### 8.3 Recommendations

We suggest that future database curators maximize the utility of their creation by making it available as a browsable website, download, and API if possible. However, we acknowledge that this is not always possible. Depositing a flat file download onto a repository hosting service is a good way to ensure that the cost of actively maintaining a browsable website does not jeopardize the longevity of the database. Other general suggestions for database creators are to cross reference protein and ligand identifiers with those of commonly used databases; to ensure that formatting is consistent, especially in downloadable files; and to keep an update log with the release date of the current version prominently displayed. These practices will make it easier for users to leverage the knowledge in the database and therefore increase the number of researchers who use it.

### 8.4 Final remarks

This review is not meant to spotlight one database as the best of all databases. The various subcategories within the list of pocket databases and the list of interaction databases are meant to illustrate that they have many different focuses and applications. We have described what makes each database distinct and the features of their contents to facilitate readers' appropriate selection of resources for their particular research endeavors.

We made every attempt to be thorough in our review of currently available databases. However, with the volume of work in the field, it is impossible to be sure that we identified every instance of relevant work. We regret any omissions and stress that these are inadvertent and not an indication of any particular opinion about any omitted databases.

## Declaration of Competing Interest

None.

## Appendix A Supplementary material

MMC 1 "Table S1: Pocket-Finding Methods". MMC 1

MMC 2 - "Table S2: Pocket and Interaction Databases". MMC 2

MMC 3 "Descriptions of the contents of Supplementary Tables 1 and 2, including specifications of the contents of each column in each table.". MMC 3
