# Supplementary material for: Databases of ligand-binding pockets and protein-ligand interactions
Source: Comput Struct Biotechnol J. 2024 Mar 24;23:1320–38. doi: 10.1016/j.csbj.2024.03.015 (PMC10997877; doi:10.1016/j.csbj.2024.03.015)
Supplement: MMC 3 — "Descriptions of the contents of Supplementary Tables 1 and 2, including specifications of the contents of each column in each table.". [file mmc3.docx]

**Table S1: Pocket-Finding Methods**

TableS1_methods.xlsx

A non-exhaustive list of pocket-finding methods. For methods referred to in the main body of the review, and some other widely used or very recent methods, the method type (e.g. geometric, ML, etc.) is provided along with links to available code or servers. For each method, we also provide all relevant papers (to the best of our knowledge).

**Table S2: Pocket and Interaction Databases**

TableS2_dbs.xlsx

Extended version of Tables 1 and 2 from the main body of the review. Includes all databases referenced in the main body and unavailable databases not referenced. Includes columns to sort databases by their groupings and subgroupings as provided in the main body. For many unavailable databases and some available databases, some information like last update or approximate size is not obtainable; this is indicated with a question mark.

Column specifications:

- Name: Name of database
- Pocket database: yes if classified as a pocket database, otherwise no
- Pocket db subcategory: Subcategory of “pocket database” as organized in main body of manuscript, if applicable
- Pocket db subsubcategory: Subcategory of subcategory of “pocket database” as organized in main body of manuscript, if applicable
- Interaction database: yes if classified as an interaction database, otherwise no
- Interaction db subcategory: Subcategory of “interaction database” as organized in main body of manuscript, if applicable
- Interaction db subsubcategory: Subcategory of subcategory of “interaction database” as organized in main body of manuscript, if applicable
- Year created: year database established or first paper published, if known
- Last updated: as of January 2024, the year in which the database was last updated, if known
- Short description: description of database
- URL: database URL
- Website available: yes if database URL directs to a loadable website with an internet browser, as of January 2024, otherwise no
- Browsable website available: yes if website available and the website allows for exploration of the database in the internet browser without downloads or API use, otherwise no
- Download available: yes if the database is available in a downloadable format, otherwise no
- Download format: file format of pocket information download, if applicable; note that other download files may be available, but for simplicity we only provide the format of the file(s) containing pocket information (or for interaction databases that aren’t pocket databases, interaction information)
- API available: yes if there is an API available from which to query the database, otherwise no
- Size: size of the database as reported on its website or most recent paper as of January 2024, if known; note that database sizes are not reported in a consistent manner due to variation in how size information is conveyed
- Known binding sites: yes if the database includes known binding sites, otherwise no
- Known binding site source: from where known binding sites are obtained, if applicable
- Predicted binding sites: yes if the database includes predicted binding sites, otherwise no
- Predicted binding site method: method for predicting binding sites, if applicable
- Binding affinity information: whether binding affinity is included in the database; values can be “yes (all)”, “yes (some”), or “no”
- Citations: *approximate* number of citations of all relevant papers as of January 2024; these values are binned to reflect that this is a low precision number (the number we calculated using Google Scholar is floored to the nearest hundred; number of citations less than 100 are indicated if they are greater than 10 or less than or equal to 10)
- Paper(s): links to all relevant papers describing the database, to the best of our knowledge, as of January 2024
